# Postoperative bile leakage caused by intrahepatic duct injury during right hemicolectomy: A case report

**DOI:** 10.1097/MD.0000000000027877

**Published:** 2021-11-19

**Authors:** Jaram Lee, Ook Song, Hyeong-Min Park, Soo Young Lee, Chang Hyun Kim, Hyeong Rok Kim

**Affiliations:** Department of Surgery, Chonnam National University Hwasun Hospital and Medical School, Hwasun, South Korea.

**Keywords:** bile peritonitis, case report, postoperative complication, right hemicolectomy

## Abstract

**Introduction:**

Bile peritonitis is one of the rare complications that can occur after cholecystectomy or hepatectomy. It is associated with high mortality, prolonged hospital stay, and increased cost. We herein report 2 cases of bile leakage as a postoperative complication of right hemicolectomy.

**Patient concerns:**

Two patients underwent a right hemicolectomy for colon cancer. Both patients had a history of cholecystectomy, and intrahepatic bile duct dilatation was observed in preoperative imaging study. During surgery, adhesiolysis was performed between the liver and the hepatic flexure of the colon due to adhesion in that area.

**Diagnosis:**

Postoperatively, bile fluid was drained via an intraabdominal drainage tube. Both cases required surgical intervention to explore the origin of the leakage. In both cases, the anastomosis was intact, and the injury of the intrahepatic bile duct just beneath the liver surface was the origin of bile leakage.

**Interventions:**

Suture ligation, irrigation, and drainage were performed in both patients.

**Outcomes:**

There was no more bile leakage after reoperation, and both patients were discharged in good health after antibiotics treatment.

**Conclusion:**

Although very rare, bile leakage due to intrahepatic duct injury can occur after right hemicolectomy in patients with a history of cholecystectomy and intrahepatic duct dilatation. It is necessary to consider the possibility of bile duct injury and anastomotic leakage if bile leakage is suspected after right hemicolectomy.

## 1 Introduction

Bile peritonitis is a serious condition with a high mortality rate of 8%–40% as it can lead to sepsis.^[1]^ Bile leakage can develop intraabdominal abscess after surgery and cause an increased hospital stay and prolonged ileus.^[2]^ The possible causes of bile leakage after surgery include injury to the bile duct or duodenum during surgery, as well as gallbladder perforation due to cholecystitis, trauma, radiofrequency ablation, or endoscopic retrograde cholangiopancreatography (ERCP).^[3]^ In rare cases, bile leakage can also be caused by spontaneous bile duct perforation.^[4–6]^

In cases of bile peritonitis after right hemicolectomy, leakage of bowel contents due to anastomotic leakage is the first to be considered as a possible cause. However, we encountered 2 cases of bile leakage caused by injury of the intrahepatic bile duct after right hemicolectomy for colon cancer.

## 2 Case presentation

### 2.1 Case 1

**Figure 1:**
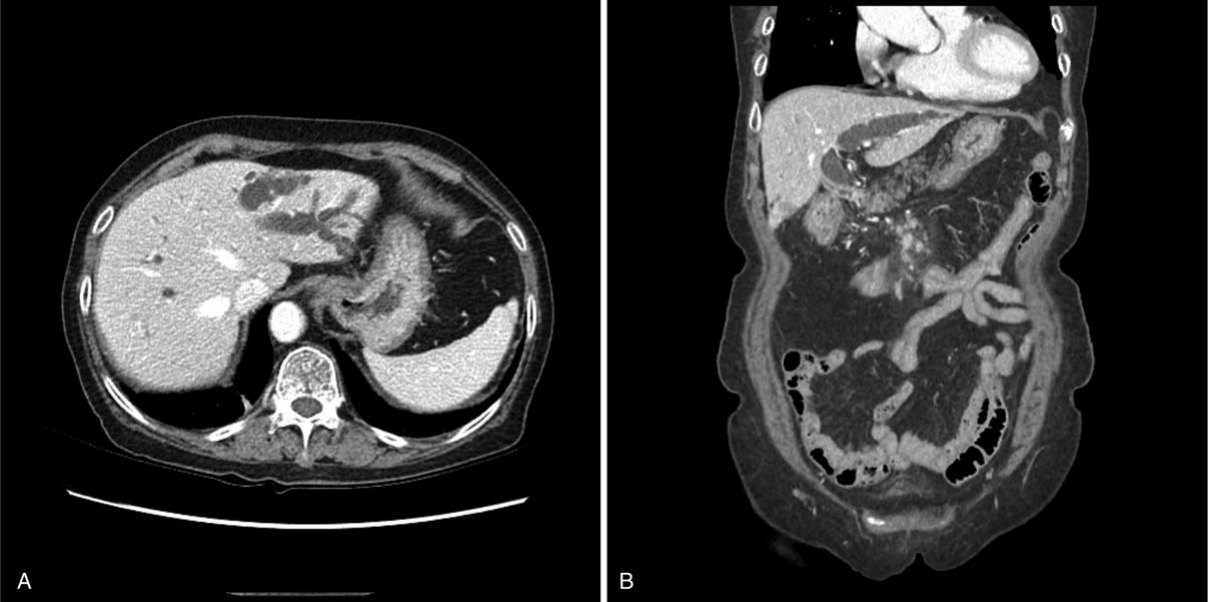
Abdominopelvic computed tomography showing dilatation of bile duct. (A) Axial view and (B) coronal view.

**Figure 2:**
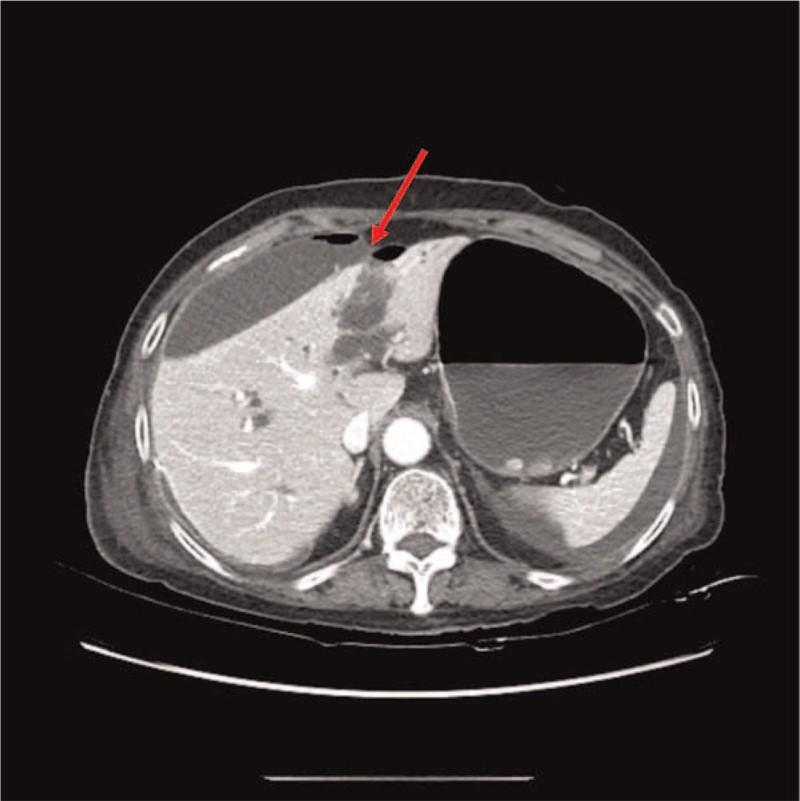
Postoperative computed tomography showing multiloculated fluid collection in perihepatic and perisplenic spaces. Red arrow indicates the point where bile leakage was found during surgery.

An 81-year-old female patient was admitted to the hospital with a complaint of epigastric pain. She was receiving medication for hypertension and had a surgical history of open cholecystectomy. An abdominopelvic computed tomography (CT) scan revealed intestinal obstruction caused by hepatic flexure colon cancer and stricture of the distal common bile duct (CBD) with upstream bile duct dilatation (Fig. 1). Magnetic resonance cholangiopancreatography was performed to discriminate distal CBD malignancy before surgery, and distal CBD stricture was considered as a benign lesion. A colonoscopic stent was inserted before surgery because of cancer obstruction. Curative resection was performed in June 2020. Laparoscopic right hemicolectomy was initially planned, however, the operation was converted to open surgery due to intraperitoneal adhesion. On postoperative day 2, laboratory values showed an increase of serum C-reactive protein (23.37 mg/dL). Her blood pressure was 90/70 mmHg and her heart rate was 103/min. Peritoneal fluid analysis showed that amylase/lipase was normal (15/8 unit/L), but total bilirubin was highly elevated (31.77 mg/dL) at more than 5 times higher than the serum level. Abdominopelvic CT showed a fluid collection in the perihepatic and perisplenic space (Fig. 2). A percutaneous drain was inserted for intraabdominal fluid collection, and 600 mL of turbid brownish fluid was initially drained. However, the nature of the drain changed into a bile color after 3 days, with an amount of 580 mL/day. With suspicion of anastomotic leakage, we performed an emergency laparotomy. The anastomosis was intact without leakage; however, bile leakage was observed on the dilated intrahepatic bile duct exposed to the liver surface (Fig. 2, arrow) where adhesiolysis was conducted during the initial surgery. After suture ligation of the liver, we confirmed that there was no further bile leakage. The patient showed satisfactory recovery and was discharged 15 days after reoperation.

### 2.2 Case 2

**Figure 3:**
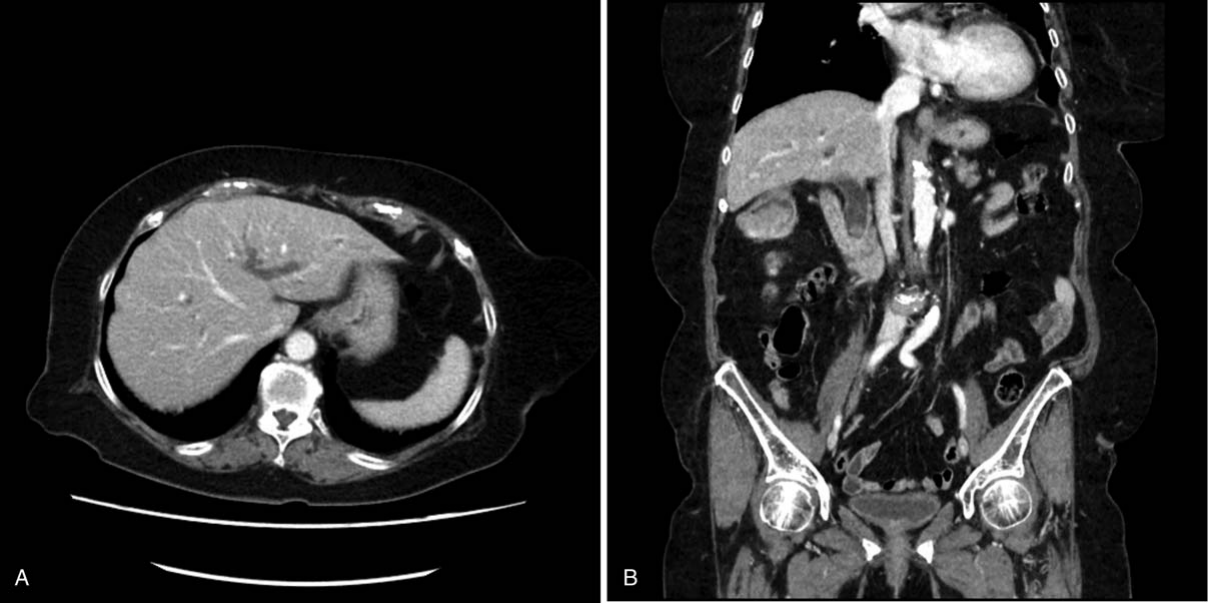
Computed tomography showing dilatation of intrahepatic duct (A) and common bile duct stone (B).

**Figure 4:**
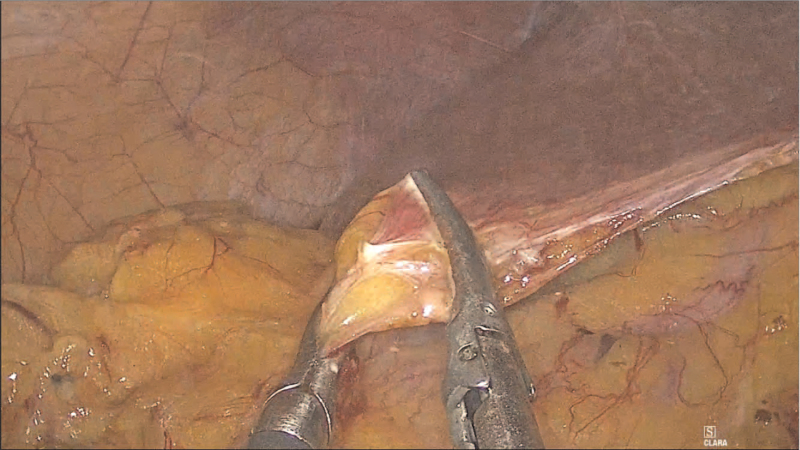
A video image during surgery showing adhesion between the liver and the hepatic flexure of the colon. It is assumed that the intrahepatic bile duct injury occurred during adhesiolysis between the liver and the omentum.

**Figure 5:**
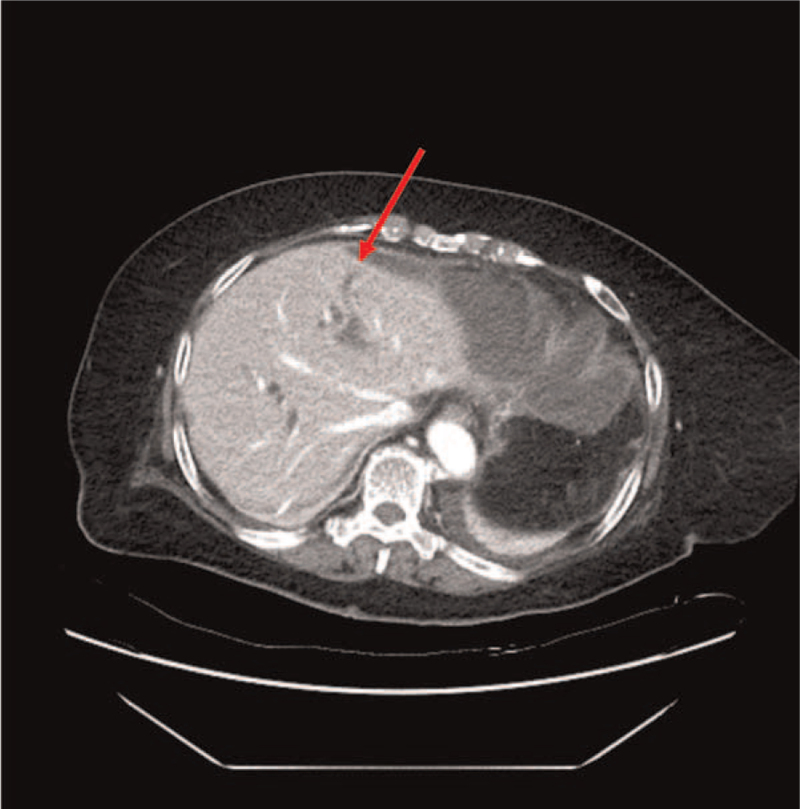
Computed tomography shows large loculated fluid collection in perihepatic space. Red arrow indicates the point where bile leakage was found during surgery.

A 78-year-old female patient was admitted for ascending colon cancer surgery. She was receiving medication for hypertension and had a surgical history of cholecystectomy and open choledocholithotomy for CBD stone. Abdominopelvic CT showed ascending colon cancer with clinical stage T3N1 and distal CBD stone with upstream bile duct dilatation (Fig. 3). However, she had no symptoms associated with bile duct dilatation, and serum bilirubin level was normal (0.74 mg/dL). Laparoscopic right hemicolectomy was performed on March 2021. There were severe adhesions because of the previous operation, and laparoscopic adhesiolysis was performed between liver surface and omentum (Fig. 4). There was a large volume of intraoperative bleeding (500 mL) due to the tearing of the middle colic vein. On postoperative day 2, 185 mL of bile fluid without foul odor was drained through the Jackson–Pratt drain. The patient complained of diffuse abdominal pain and had tenderness on her right upper abdomen. Her body temperature was elevated to 38.3°C and heart rate was 116 beats/min. Peritoneal fluid laboratory tests showed an increase of total bilirubin (30.43 mg/dL). On abdominopelvic CT, the anastomosis was intact, but fluid collection around the liver was observed (Fig. 5). Considering the possibility of CBD or duodenum injury and anastomotic leakage, the patient underwent emergency surgery. The anastomosis was intact without leakage, and there was no injury to the duodenum or CBD. However, a pinpoint opening of the intrahepatic bile duct was observed at the edge of the liver surface where adhesiolysis was performed during initial surgery due to omental adhesion (Fig. 4). There was bile leakage through the injured bile duct (Fig. 5, arrow). Suture ligation and clipping were performed, and the operation was finished after confirming no further bile leakage. The patient recovered without additional complications and was discharged in good condition 9 days after reoperation. She will receive ERCP for distal CBD stone via the gastroenterology outpatient clinic.

## 3 Discussion and conclusions

Bile peritonitis can occur for various reasons. Cholecystectomy and hepatectomy are the most common causes of postoperative bile leakage. In rare cases, it can also be caused by upper gastrointestinal surgery.^[3,7,8]^ However, bile leakage after right hemicolectomy is extremely rare with only a few reports to date. In the present cases, bile leakage was confirmed through peritoneal fluid analysis, which revealed that the peritoneal bilirubin level was 5 times higher than serum level, and that right upper quadrant pain, leukocytosis, and elevated C-reactive protein was observed. When these findings were observed after right hemicolectomy, anatomic leakage was considered, and, therefore, it was very difficult to suspect bile leakage due to intrahepatic duct injury as in the present cases.

It is of note that intrahepatic bile duct dilatation was observed due to CBD stricture or CBD stones on preoperative imaging study in both patients. A previous study showed that the presence of stones in the distal CBD may cause spontaneous bile duct perforation due to an increase in biliary pressure.^[6]^ An acute attack of cholangitis, biliary enteric anastomosis, and previous hepatectomy or biliary surgery within 1 month of the surgery were reported to be independent risk factors for bile leakage.^[9]^ In our cases, the pressure on the bile duct was inevitably high, and the intrahepatic bile duct was dilated to the very end of the liver surface so that bile leakage could easily occur even with a minor liver surface injury. Therefore, when the intrahepatic bile duct was dilated, and intraabdominal adhesion was suspected due to the previous history of surgery, such as cholecystectomy, we should pay more attention to avoid liver injury during adhesiolysis and perform meticulous dissections. In addition, the possibility of bile leakage can be reduced by reducing the bile duct pressure through stone removal or tube insertion through ERCP before surgery.^[10]^

When the liver injury was suspected during surgery in patients with bile duct dilatation as in the present cases, bile leakage can be prevented by suture ligation during surgery. A previous study reported that the bilirubin concentration in the drain was low during the early postoperative period when fibrin glue was applied to the cut surface after hepatectomy.^[11]^ Therefore, when the liver injury was suspected during surgery without definite evidence of bile leakage, applying fibrin glue or sealant on the liver surface can be another option to prevent bile leakage.

In the present cases, after diagnosis of bile leakage, emergency surgery with suture ligation of the bile duct was performed. Most of the bile leakage after hepatectomy or cholecystectomy was known to be improved by non-surgical treatment, such as percutaneous drainage, ERCP, and biliary stent insertion.^[12,13]^ However, it is questionable whether non-surgical treatment, such as maintenance of drainage tubes, can improve bile leakage in the present cases, with an amount of >150 mL/day. Furthermore, in our cases, the probability of anastomotic leakage had to be considered first, making it difficult to perform conservative treatment without surgical exploration. If the possibility of anastomotic leakage is overlooked, it may lead to fatal consequences, such as septic shock. By rapid re-laparotomy, both patients were able to recover quickly and thus receive adjuvant chemotherapy without delay.

In summary, although very rare, bile leakage from intrahepatic duct injury can occur after right hemicolectomy, especially in patients with CBD stricture or CBD stones and history of the previous cholecystectomy. In such patients, rapid surgical intervention is required to distinguish the possibility of bile duct injury and anastomotic leakage. If intrahepatic duct dilatation was observed in the preoperative imaging study, reducing biliary pressure through preoperative intervention and performing meticulous dissection during surgery may help reduce bile leakage.

## Author contributions

JL, SYL, and HRK are the operating surgeons and participated in the preparation of the manuscript. HMP and OS participated in patient data collection and image revisions, HMP and CHK were involved in patient care. JL and SYL wrote the manuscript. All authors read and approved the final case report.

**Conceptualization:** Jaram Lee, Soo Young Lee, Chang Hyun Kim, Hyeong Rok Kim.

**Data curation:** Jaram Lee, Ook Song, Hyeong-Min Park, Soo Young Lee, Chang Hyun Kim, Hyeong Rok Kim.

**Formal analysis:** Jaram Lee, Ook Song, Soo Young Lee.

**Investigation:** Jaram Lee, Hyeong-Min Park, Soo Young Lee.

**Methodology:** Jaram Lee, Hyeong-Min Park, Soo Young Lee.

**Resources:** Jaram Lee.

**Supervision:** Soo Young Lee.

**Writing – original draft:** Jaram Lee, Soo Young Lee.

**Writing – review & editing:** Jaram Lee, Ook Song, Hyeong-Min Park, Soo Young Lee, Chang Hyun Kim, Hyeong Rok Kim.

## References

[1] Andersson R Tranberg KG Bengmark S. Roles of bile and bacteria in biliary peritonitis. Br J Surg 1990;77:36–9. 2405932 10.1002/bjs.1800770113

[2] Spetzler VN Schepers M Pinnschmidt HO Fischer L Nashan B Li J. The incidence and severity of post-hepatectomy bile leaks is affected by surgical indications, preoperative chemotherapy, and surgical procedures. Hepatobiliary Surg Nutr 2019;8:101–10. 31098357 10.21037/hbsn.2019.02.06 PMC6503260

[3] Kapoor S Nundy S. Bile duct leaks from the intrahepatic biliary tree: a review of its etiology, incidence, and management. HPB Surg 2012;2012:752932. 22645406 10.1155/2012/752932 PMC3356893

[4] Shukla RM Roy D Mukherjee PP. Spontaneous gall bladder perforation: a rare condition in the differential diagnosis of acute abdomen in children. J Pediatr Surg 2011;46:241–3. 21238677 10.1016/j.jpedsurg.2010.09.043

[5] Cai W Pan K Li Q Miao X Shu C. Biliary peritonitis due to spontaneous perforation of the left intrahepatic bile duct in an adult: a case report and review of literature. Int Surg 2018;103:339–43.

[6] Kang SB Han HS Min SK Lee HK. Nontraumatic perforation of the bile duct in adults. Arch Surg 2004;139:1083–7. 15492148 10.1001/archsurg.139.10.1083

[7] Tanaka S Hirohashi K Tanaka H. Incidence and management of bile leakage after hepatic resection for malignant hepatic tumors. J Am Coll Surg 2002;195:484–9. 12375753 10.1016/s1072-7515(02)01288-7

[8] Koch M Garden OJ Padbury R. Bile leakage after hepatobiliary and pancreatic surgery: a definition and grading of severity by the international study group of liver surgery. Surgery 2011;149:680–8. 21316725 10.1016/j.surg.2010.12.002

[9] Zhang GW Lin JH Qian JP Zhou J. Analyzing risk factors for early postoperative bile leakage based on clavien classification in bile duct stones. Int J Surg 2014;12:757–61. 24909135 10.1016/j.ijsu.2014.05.079

[10] El-Gendi AM El-Shafei M Bedewy E. The role of prophylactic endoscopic sphincterotomy for prevention of postoperative bile leak in hydatid liver disease: a randomized controlled study. J Laparoendosc Adv Surg Tech A 2018;28:990–6. 29641366 10.1089/lap.2017.0674

[11] Capussotti L Ferrero A Viganò L Sgotto E Muratore A Polastri R. Bile leakage and liver resection. Arch Surg 2006;141:690–4. 16847242 10.1001/archsurg.141.7.690

[12] Giri S Sundaram S Darak H Kumar S Bhatia S. Outcomes of endoscopic management among patients with bile leak of various etiologies at a tertiary care center. Clin Endosc 2020;53:727–34. 32819052 10.5946/ce.2020.017 PMC7719417

[13] Stampfl U Hackert T Radeleff B. Percutaneous management of postoperative bile leaks after upper gastrointestinal surgery. Cardiovasc Intervent Radiol 2011;34:808–15. 21301846 10.1007/s00270-011-0104-3

